# Recurrent 6^th^ nerve palsy in a child following different live attenuated vaccines: case report

**DOI:** 10.1186/1471-2334-12-105

**Published:** 2012-04-30

**Authors:** Daryl R Cheng, Nigel W Crawford, Michael Hayman, Christopher Buckley, Jim P Buttery

**Affiliations:** 1 Central Medical School, Faculty of Medicine, Nursing & Health Sciences, Monash University, Melbourne, Australia; 2 SAEFVIC, Murdoch Children’s Research Institute (MCRI), Melbourne, Australia; 3 Department of General Medicine, Royal Children's Hospital (RCH), Melbourne, Australia; 4 Department of Pediatrics, The University of Melbourne, Melbourne, Australia; 5 Department of Neurology, Royal Children’s Hospital (RCH), Melbourne, Australia; 6 Department of Pediatric Neurology, Monash Children’s, Melbourne, Australia; 7 Department of Pediatrics, Monash University, Melbourne, Australia; 8 Department of Surgery, Southern Health, Melbourne, Australia; 9 Pediatric Infectious Diseases Unit, Monash Children's, Melbourne, Australia

**Keywords:** Pediatric, Immunization, 6^th^ nerve, Palsy, Vaccine

## Abstract

**Background:**

Recurrent benign 6^th^ nerve palsy in the paediatric age group is uncommon, but has been described following viral and bacterial infections. It has also been temporally associated with immunization, but has not been previously described following two different live attenuated vaccines.

**Case presentation:**

A case is presented of a 12 month old Caucasian boy with recurrent benign 6^th^ nerve palsy following *measles-mumps-rubella* and varicella vaccines, given on separate occasions with complete recovery following each episode. No alternate underlying etiology was identified despite extensive investigations and review.

**Conclusions:**

The majority of benign 6th nerve palsies do not have a sinister cause and have an excellent prognosis, with recovery expected in most cases. The exact pathophysiology is unknown, although hypotheses including autoimmune mechanisms and direct viral invasion could explain the pathophysiology behind immunization related nerve palsies. It is important to rule out other aetiologies with thorough history, physical examination and investigations. There is limited information in the literature regarding the safety of a repeat dose of a live vaccine in this setting. Future immunizations should be considered on a case-by-case basis.

## Background

The age and gender-adjusted incidence of sixth nerve palsy is 11.3 per 100,000, with 26% of cases with undetermined or benign aetiology [1]. Within the paediatric age group, acute benign sixth nerve palsy accounts for 9-14% of all sixth nerve palsies [2]. It is of presumed inflammatory etiology, and may follow viral and bacterial infections such as *Varicella zoster*, Epstein-Barr, Cytomegalovirus, *Staphylococcus aureusCoxiella burnetti* and *Borrelia burgdorferi*[3-6]. Recurrent palsies are rarer still, with presumed similar etiology. Recurrence has been temporally associated with immunization [4], including both inactivated and live attenuated viral vaccines (Table 1). It has not previously been described following two different live attenuated vaccines. A case of recurrent sixth nerve palsy following different live attenuated vaccines is reported and discussed.

**Table 1:** Clinical characteristics of patients with benign 3^rd^ and 6^th^ nerve palsy post-immunization

**Authors**	**Age/Gender/Side of paralysis**	**Vaccine Type**	**CN**	**Time-post Vaccine**	**Investigations**	**Outcome**
Werner *et al.*[5]	1 yr 3 mths/F/L	MMR	VI	10 days	CT Brain	Resolved over 3 months
	0 yr 8 mths/F/L	DPT	VI	6 weeks	Nil	Resolved over 6 months
		DPT Booster	VI	3mths	Nil	Resolved over 2 months
McCormick *et al.*[7]	1 yr 1 mth/F/L	MMR	VI	1 week	FBE, CRP, Anti-Nuclear Antibody (ANA), Measles and mumps serum titers, Anti-Mitochondrial antibody, blood cultures, serum rotavirus titer	Resolved over 8 weeks
					Feces culture for parasites and *C difficile*	
					CT Brain	
Leidermann *et al.*[8]	1 yr 5 mths/M/R	Influenza	VI	3 weeks	MRI Brain (T1, T2, Flair)	Initial 10–20 degree incomitant esotropia with limitation of abduction
						12 weeks – 10 degree esotropia in R gaze
						7 months – residual motility deficit
Sturm *et al.*[3]	6 mths/M/R	DPT, Polio, Hib	VI	6 months	MRI Brain	Resolved at 5 days
Mahoney *et al.*[9]	1 yr 8 mths/M/L	DPT, Hib	VI	3 weeks	MRI, AchRab	Improving but incomplete
	2 yr 7 mths/M/L	VZV	VI	2.5 weeks		
Manzotti *et al.*[10]	20mths/M/L	MMR	III	3 weeks	FBE, UEC	Resolved at 20 days
					CMV, EBV, HSV,	
					VZV, Parvo B19, Influenza, Parainfluenza, RSV titers	
					Electroencephalography, MRI brain	
Chan *et al.*[11]	1 yr 5 mths/M/R	Edmonston Measles	III	2 weeks	FBE, UEC, ESR, blood sugar level, CSF for protein, glucose, viral, bacterial, Blood cultures, Serum measles titer	Resolved at 2 months
					Skull x-ray, electroencephalography, CT Brain	
					PPD Tuberculin Skin test	
					Intravenous Pyelogram (IVP)	
					Bone marrow aspirate	

*FBE* full blood examination, *UEC* urea, electrolytes, creatinine, *CRP* C-reactive protein, *CT* computed tomography, *MRI* magnetic resonance imaging, *CMV* cytomegalovirus, *EBV* Epstein-Barr virus, *HSV* herpes simplex virus, *VZV* varicella zoster virus, *RSV* respiratory syncytial virus, *DTPdiphtheria-tetanus –pertussis*; *MMR measles-mumps-rubella; Hib Haemophilus influenzae type b, AchRab* acetylcholine receptor antibody testing.

## Case presentation

A previously healthy 12 month old boy presented to hospital with a right 6^th^ nerve palsy seven days after his routine 12 month immunizations: measles, mumps, rubella (MMR) (Priorix®, *GSK)* and *Haemophilus influenzae* type b (Hib) – Hepatitis B (Comvax®, *Merck).* He had been well for the first four days post-immunization, but then noted by his grandparents to have a “squint” and appear lethargic. He presented for medical attention on day seven post-vaccination because of persistent concerns about his unusual eye movements.

Two months earlier he had received his second catch-up dose of 7-valent pneumococcal conjugate vaccination (Prevenar®, *Wyeth Vaccines*) and meningococcal group C conjugate vaccine (Meningitec®, *Wyeth Vaccines*). He had experienced no adverse events from any of his previous vaccinations, which were up to date for his age on the routine Australian National Immunization Program schedule [12]. He had a past history of emergency delivery at 36 weeks gestation by caesarean section for twin-twin transfusion following a twin pregnancy conceived by *in-vitro* fertilization. There were no significant neonatal problems and he was developmentally age appropriate.

On admission he was afebrile, blood pressure 117/59, pulse rate 120/min, respiratory rate 30/min and oxygen saturation of 95%. There was no history of preceding viral illness or prodrome and no symptoms developed during admission. Further evaluation by a pediatric neurologist and ophthalmologist confirmed an isolated right 6^th^ nerve palsy, with no evidence of facial asymmetry. There was mild plagiocephaly, with a head circumference of 46.5 cm (50^th^ centile). No ptosis was evident, and fundoscopic examination bilaterally was normal.

Initial blood investigations included normal full blood examination (Hb 128 g/L; Platelets 284 x10^9^/L; leukocytes 11.1 x 10^9^/L and normal baseline electrolytes, liver function tests, creatinine kinase (CK) and serum calcium levels. The erythrocyte sedimentation rate (ESR) was normal (3 mm/hr).

Neuroimaging with magnetic resonance imaging (MRI) and angiography (MRA) of the brain demonstrated no intracranial abnormality. Cerebrospinal fluid (CSF) examination revealed no white cells, 89 red cells x10^6^/L, protein of 0.19 g/L (0.20-0.40), glucose 2.8 mmol/L (2.8-4.0) and lactate of 1.2 mmol/L. A random blood glucose level was 6.0 mmol/l. There was no growth on CSF cultures. The CSF opening pressure was 31 cm CSF, but this was obtained at the third attempt at a lumbar puncture in a screaming child, and the pressure was therefore likely artefactually elevated. Acetylcholine receptor antibodies were negative (0.2 nmol/L).

The differential diagnoses with this presentation included a post viral 6^th^ nerve palsy, idiopathic cranial nerve palsy, myasthenia gravis and benign intracranial hypertension. He was initially trialed on Pyridostigmine (Mestinon® *Valeant)* 5 mg three times daily. This was subsequently increased to 10 mg three times daily, but with no change in clinical signs it was ceased. The 6^th^ nerve palsy persisted at one-week post vaccination, but completely resolved spontaneously over the next six weeks, with resolution confirmed on ophthalmology follow-up.

The child then re-presented aged 20 months with a recurrent episode of a right 6^th^ nerve palsy. On this occasion it commenced followed a different live virus vaccination – Varicella (Varivax® *Merck*) given four weeks earlier. Again, there was no viral prodrome and he had otherwise been alert and well. He presented with identical symptoms of a squint and was brought to hospital for evaluation five weeks after immunization. Repeat examination by an ophthalmologist and pediatric neurologist again confirmed an isolated 6^th^ nerve palsy with no evidence of papilledema. He was afebrile and haemodynamically stable. The nerve palsy worsened over the subsequent two days, then significantly improved over the next 7–10 days and had entirely resolved by 5 weeks, now a total of 9 weeks post immunisation. On this presentation, he did not have any neuro-imaging, CSF analysis or repeat blood investigations.

Over the next 12 months the child tolerated numerous viral infections with no recurrence of the squint or any neurological symptoms. He had ongoing ophthalmology review with no abnormalities noted. A follow-up with pediatric neurology at two years of age confirmed a normal examination, with all appropriate developmental milestones reached.

Following detailed discussion with the family it was advised that further live attenuated vaccines not be administered, even though the routine Australian immunization schedule includes a MMR vaccine (2^nd^ dose) at 4 years of age [12]. The main reason for this advice was that no previous recurrent cases had been reported in the scientific literature and therefore the safety of further live attenuated vaccines in this setting was unknown.

## Discussion

By definition, a benign 6^th^ nerve palsy is not due to a sinister underlying cause, such as an underlying space occupying lesion, and recovery is expected. The condition has a female predominance and the left side is most commonly affected, in contrast to this case [13].

The condition is a known sequel of viral illnesses, infections and immunization [4]. The majority of palsies described as associated with immunization have involved attenuated live vaccinations, including MMR and VZV (see Table 1) [13]. To our knowledge, this is the first documented case of recurrent benign 6^th^ nerve palsy following two different live attenuated vaccinations.

Whilst an isolated 6^th^ cranial nerve palsy may seem less likely to be due to an underlying sinister cause, up to a third of such palsies in children have a neoplastic origin [14]. Therefore, a thorough history and physical examination is recommended, in combination with an MRI brain scan [15]. Fasting blood glucose, complete cell count, blood pressure evaluation, lumbar puncture and other investigations may be warranted, but should be considered on a case-by-case basis. Further review by a neurologist and ophthalmologist is recommended to assess if additional symptoms or clinical signs arise over time.

Despite a seemingly temporal association between benign 6^th^ nerve palsy and immunization in children, the pathophysiological mechanism and exact site of cellular injury remains unclear. Hypotheses include damage from autoimmune mediation or direct viral invasion causing demyelination, localized arteritis or genetic predisposition, which could increase susceptibility to such nerve palsies [5,16].

The hypothesis that it could relate to additives in the vaccine is unlikely. Neomycin is used as a preservative to prevent bacterial contamination in both the MMR and varicella vaccines, but has only been shown to contribute to local or systemic allergic anaphylactic reactions [17]. It is also an additive in many of the non-live vaccines the patient received in childhood without incident and extensive epidemiological studies have not found a link between neurological adverse events following immunization (AEFI) and additives or adjuvants in vaccines [18].

Autoimmune mechanisms post-vaccination may help explain recurrent mononeuritis AEFI. Mutsch *et al.* proposed an autoimmune cause from vaccine adjuvants amongst other etiologies for Bell’s palsy associated with an inactivated intra-nasal influenza vaccine [19]. Clifford *et al.* reported three cases of orchitis post-measles mumps rubella vaccine, which were possibly auto-immune related given a short-time course to development of inflammation [20].

The typical incubation period for wild type measles infection is approximately 10 days (range 7–18 days); attenuation has been shown to increase the incubation period for some vaccines including measles [21]. This is also reflected in varicella infections, where development of antibodies post vaccination to vaccine strain virus appear later compared to those in wild type infection (range 10–21 days for wild type) [22]. Mumps and rubella have incubation periods of 14–25 days and 12–23 days respectively [12]. Our patient’s incubation period was 5 days after MMR vaccine and 28 days after varicella vaccine in his two recurrent episodes, which therefore could possibly be the result of a vaccine strain infection invading and affecting the nerve root.

In adults, nerve palsies can be secondary to an ischemic mononeuropathy [23], but there was no evidence of this on MRI and is unlikely in the pediatric age group. They can also be triggered by inflammatory conditions such as systemic lupus erythematous (SLE), but again there was no evidence of such an etiology in this case, with a normal ESR and no CSF pleocytosis.

Although the Hib-Hepatitis B vaccine was also administered, the delayed onset (7 days) makes Hib or Hepatitis B causation of the patient’s symptoms very unlikely and less biologically plausible. As it is an inactivated vaccine, one would expect to see most reactions occur within 24 hours post immunisation.

There is limited information in the literature regarding the safety of a repeat dose of a live vaccine in this setting. As detailed above, a recurrent case of a nerve palsy has been described post MMR [5,7]. A second and further dose is recommended to increase the likelihood of sero-conversion, but ultimately further immunizations should be considered on an individual basis. If travel is planned to a country endemic with measles, mumps or rubella, serological assessment could be undertaken to determine adequate sero-conversion. Within Australia however, where endemic measles is eliminated, and the rates of mumps and rubella are 0.6 and 0.15 per 100,000 per year respectively [12], the risk of non-immunization is relatively low. Whilst in this case it was felt appropriate to not administer any further live-attenuated vaccines, it was recommended to continue scheduled non-live vaccines, including *diphtheria-tetanus-acellular pertussis* (dTap).

## Conclusions

In general, prognosis for benign recurrent 6^th^ nerve palsy is excellent. The majority of patients recover full muscle function and have resolution of all symptoms [14]. Benign 6^th^ nerve palsy post-immunization is a rare occurrence that generally resolves spontaneously but needs to be thoroughly investigated and followed-up to ensure best outcome. The exact pathophysiology is unknown but could be related to autoimmune mechanisms or local demyelination. Future immunizations should be considered on a case-by-case basis.

## Consent

Written informed consent was obtained from the patient’s parents for publication of this Case report and any accompanying images. A copy of the written consent is available for review by the Series Editor of this journal.

## Abbreviations

MMR = Measles, Mumps, Rubella; Hib = Haemophilus influenzae b; AEFI = Adverse events following immunization; dTap = Diphtheria-tetanus-acellular pertussis.

## Competing interests

There were no funding sources for this paper. NWC and JPB have acted as chief investigators for epidemiological studies sponsored by vaccine manufacturers (CSL) and serological testing (Merck). All payments, including for sitting on advisory boards (NWC), data safety monitoring boards (JPB), lecturing (NWC) and travel expenses for attendance at scientific meetings, are paid directly to an administrative fund held by Murdoch Children’s Research Institute.

## Authors’ contribution

All authors have made substantial contributions in the drafting, editing, writing and revising of this article. DC and NC have coordinated the write-up of the manuscript and are responsible for final edits of the article. All authors have read and approved the final version.

## Pre-publication history

The pre-publication history for this paper can be accessed here:

http://www.biomedcentral.com/1471-2334/12/105/prepub
